# Senescence-associated secretory phenotypes in mesenchymal cells contribute to cytotoxic immune response in oral lichen planus

**DOI:** 10.1186/s12979-023-00400-5

**Published:** 2023-12-05

**Authors:** Shogo Ijima, Yuki Saito, Sena Yamamoto, Kentaro Nagaoka, Taiki Iwamoto, Arisa Kita, Maki Miyajima, Tsukasa Sato, Akihiro Miyazaki, Takako S. Chikenji

**Affiliations:** 1 Department of Oral Surgery, Sapporo Medical University School of Medicine, Sapporo, 060-8556 Japan; 2 Department of Anatomy, Sapporo Medical University School of Medicine, Sapporo, 060-8556 Japan; 3 Graduate School of Health Sciences, Hokkaido University, Sapporo, 060-0812 Japan

**Keywords:** Oral lichen planus, Senescence, Senescence-associated secretory phenotype, Mesenchymal cells, Gene set enrichment analysis, Single-cell RNA sequencing analysis

## Abstract

Oral lichen planus is a chronic inflammatory condition that adversely affects the oral mucosa; however, its etiology remains elusive. Consequently, therapeutic interventions for oral lichen planus are limited to symptomatic management. This study provides evidence of the accumulation of senescent mesenchymal cells, CD8 + T cells, and natural killer cells in patients with oral lichen planus. We profiled the patients’ tissues using the National Center for Biotechnology Information Gene Expression Omnibus database and found that senescence-related genes were upregulated in these tissues by gene set enrichment analysis. Immunohistochemical analysis showed increased senescent mesenchymal cells in the subepithelial layer of patients with oral lichen planus. Single-cell RNA-seq data retrieved from the Gene Expression Omnibus database of patients with oral lichen planus revealed that mesenchymal cells were marked by the upregulation of senescence-related genes. Cell-cell communication analysis using CellChat showed that senescent mesenchymal cells significantly influenced CD8 + T cells and natural killer cells via CXCL12-CXCR4 signaling, which is known to activate and recruit CD8 + T cells and NK cells. Finally, in vitro assays demonstrated that the secretion of senescence-associated factors from mesenchymal cells stimulated the activation of T cells and natural killer cells and promoted epithelial cell senescence and cytotoxicity. These findings suggest that the accumulation of mesenchymal cells with senescence-associated secretory phenotype may be a key driver of oral lichen planus pathogenesis.

**Supplementary Information:**

The online version contains supplementary material available at 10.1186/s12979-023-00400-5.

## Introduction

Oral lichen planus (OLP) is a persistent inflammatory condition that affects the oral mucosa [1]. It manifests as a painful erythematous center with erosions, ulcerations, and a painless or less painful white variant with papular or reticular patches on the cheeks, lips, tongue, and palate [1]. OLP has a prevalence rate of 0.38–2.05% in the general population [2], with a higher prevalence in middle-aged females [1–3]. The etiology of OLP remains poorly understood, although various factors have been postulated, including genetics, dental materials, medications, infectious agents, autoimmune disorders, immunodeficiency, dietary factors, allergies, stress, habits, trauma, diabetes, hypertension, cancer, and gastrointestinal diseases [4, 5]. Therefore, the current treatment for OLP primarily focuses on symptom relief rather than a definitive cure. A better understanding of the underlying pathogenesis of OLP is crucial to develop novel therapeutic targets [6].

Cellular senescence is characterized by irreversible cell cycle arrest, which can be triggered by many factors, including DNA damage, telomere dysfunction, oncogene activation, and organelle stress [7, 8]. The main physiological purpose of cellular senescence is to prevent the proliferation of damaged cells and trigger tissue repair by secreting various soluble molecules, a phenotype termed senescence-associated secretory phenotype (SASP) [7, 8]. The list of these molecules is not comprehensive, and the molecules can vary based on cell type and triggering factors but usually include interleukins, chemokines, growth factors, metalloproteinases, and other insoluble proteins and extracellular matrix components [7–9]. However, aging or persistent damage causes the accumulation of senescent cells and impairs cell removal by the immune system, leading to the accumulation of chronic senescent cells and the promotion of chronic inflammatory pathologies [10, 11].

Histopathologically, OLP tissues exhibit a band of T lymphocytes that infiltrate the lamina propria and cytoid bodies in the epithelial layer [3, 12]. Both CD4 + helper T cells and CD8 + cytotoxic T cells are involved in OLP. The activated helper T cells can secrete interleukin (IL)-2 and interferon (IFN)-γ, which activate the cytotoxic T cells and promote their proliferation [4]. Activated cytotoxic T cells can trigger the apoptosis of basal keratinocytes and result in the liquefaction degeneration of basal epithelial cells, which are typically found in OLP lesions and contribute to the chronic inflammation observed in OLP [13]. Natural killer (NK) cells and T cells originate from lymphoid progenitor cells and share developmental proximity. NK cells have cytolytic functions that resemble those of CD8 + cytotoxic T lymphocytes^14^. The CD56^high^CD16^−^ NK cell subset found in cutaneous lichen planus (LP) lesions are highly positive for perforin and natural cytotoxic receptors NKG2D and NKp44 [14], suggesting that NK cells are involved in LP pathology [14]. Although cutaneous LP and OLP are distinguished by a marked heterogeneity of clinical course, both share similar histological features. Therefore, both cytotoxic CD8 + T cells and NK cells are speculated to contribute to OLP pathology [12, 15].

Although SASP attracts various immune cells, including NK and CD8 + T cells [16], to sites of chronic inflammation, studies on senescence and the SASP in OLP are limited. Previous studies have reported an increase in the expression levels of NF-kappa B-associated cytokines, such as IL-1 A, IL-6, IL-8, and TNF-alpha, in the saliva and oral fluid of patients with OLP [17, 18]. These inflammatory cytokines are secreted by senescent cells as significant components of the SASP in chronic inflammatory pathology in various organs [19–22]. In addition, several studies have reported that the number of p21^CIP1/WAF1^, p16^INK4A^, and p53-positive cells, which are cell senescence biomarkers, was increased in the epithelial layer of patients with OLP [23–25]. Based on these findings, a hypothesis was proposed regarding an association between senescence and OLP.

Here, we show that senescence-related genes are enriched in the tissues of patients with OLP based on gene set enrichment analysis (GSEA) results. Single-cell RNA sequencing (scRNA-seq) and immunohistochemical (IHC) analysis showed that mesenchymal cells upregulate senescence-related genes in patients with OLP, which accumulate in the subepithelial layer that is heavily infiltrated by CD45 + immune cells. Cell-cell communication analysis using scRNA-Seq revealed that senescent mesenchymal cells significantly influenced CD8 + T cells and NK cells via CXCL12-CXCR4 signaling. Lastly, in vitro experiments demonstrated that SASP from senescent fibroblasts enhances the activation and proliferation of NK cells and T cells and induces senescence and cytotoxicity in epithelial cells.

## Results

### Expression of cellular senescence-related gene sets was enhanced in patients with OLP

**Fig. 1:**
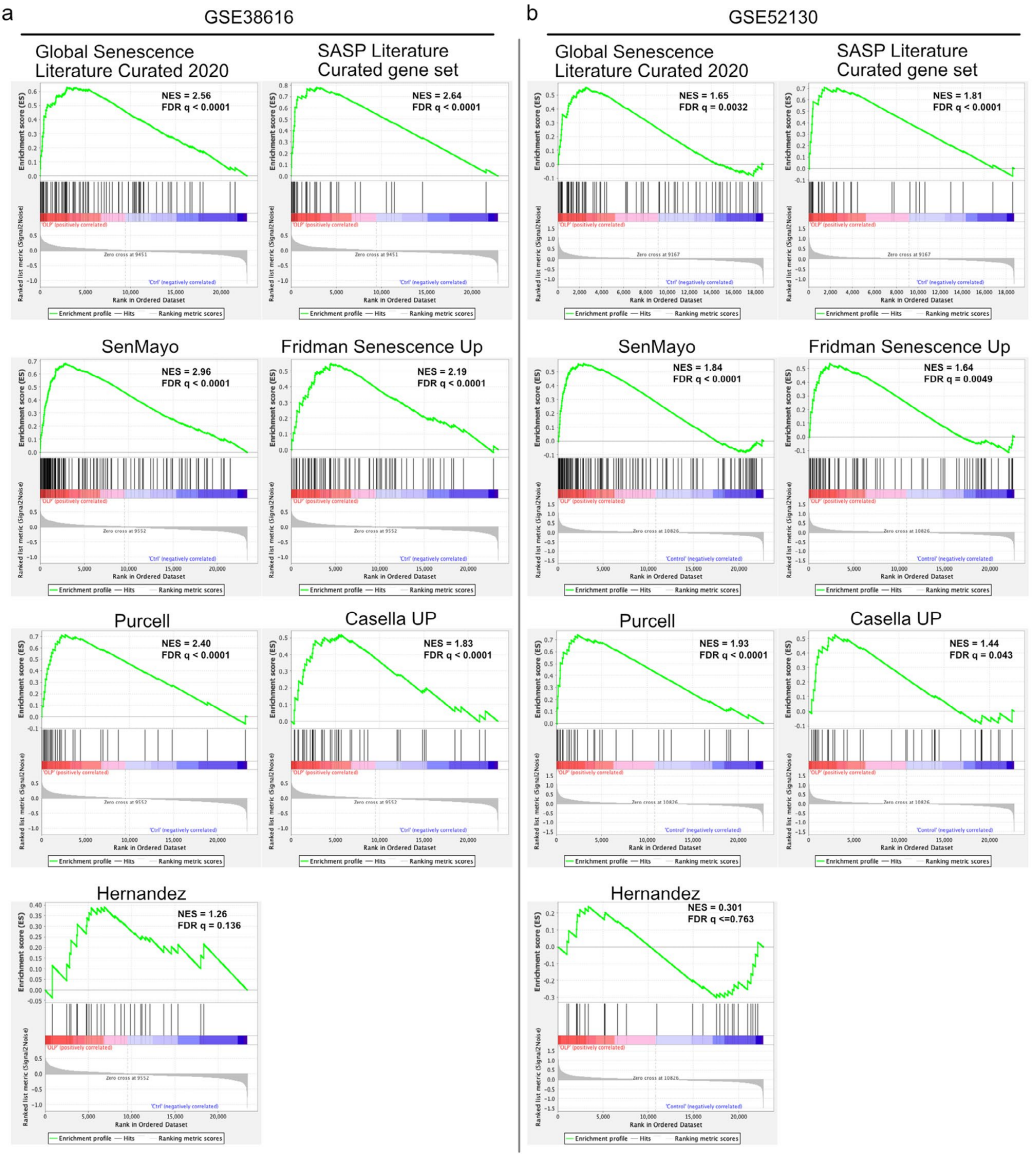
Senescence-related gene set enrichment analysis in patients with oral lichen planus and control subjects. Gene set enrichment analysis (GSEA) was performed for six gene sets related to senescence and senescence-associated secretory phenotype, including the Global Senescence Literature Curated 2020, SASP Literature Curated gene set, SenMayo, Fridman_Up, Purcell, Cassela_Up, and Hernandez, in patients with oral lichen planus and control subjects from the Gene Enrichment Omnibus database ((**a**) GSE38616 and (**b**) GSE52130). The normalized enrichment scores and false discovery rate q-values are listed in each GSEA plot

To explore whether cellular senescence contributes to OLP, we performed GSEA using the GSE38616 and GSE52130 datasets available in the National Center for Biotechnology Information (NCBI) Gene Expression Omnibus (GEO) database. We used several gene sets related to cellular senescence, including the Global Senescence Literature Curated 2020, SASP Literature Curated gene set, SenMayo, Fridman_Up, Purcell, Casella_Up, and Hernandez [26–31]. GSEA showed that OLP patient-derived oral mucosa (GSE38616) and oral epithelium (GSE52130) were significantly enriched in the Global Senescence Literature Curated 2020, SASP Literature Curated gene set, SenMayo, Fridman_Up, Purcell, and Casella_Up (Fig. 1a).

### Senescent mesenchymal cells were increased in patients with OLP

**Fig. 2:**
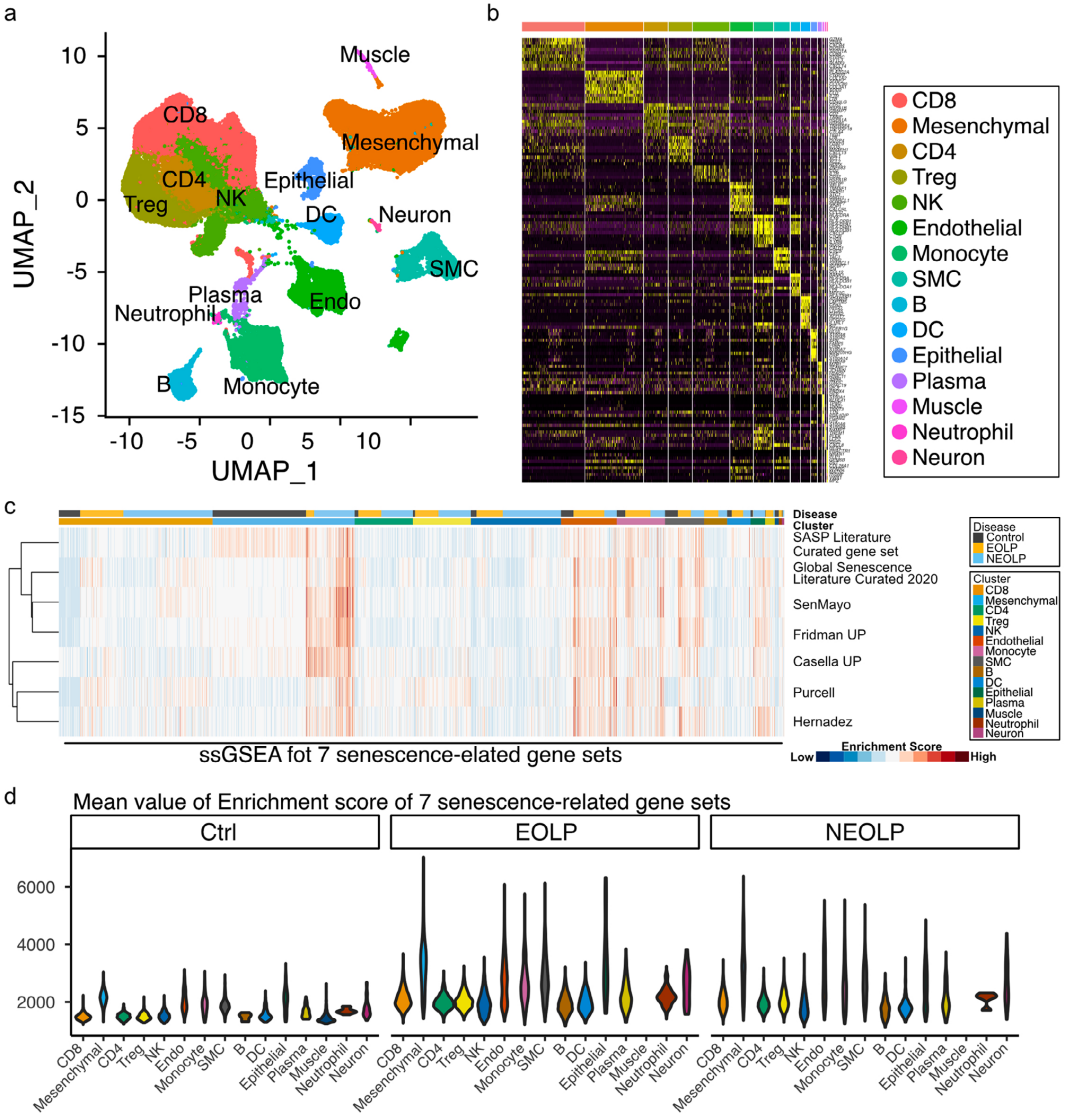
Mesenchymal cells had increased senescence-related gene expression in oral lichen planus. (**a**) UMAP plot of buccal mucosa cells from control, EOLP, and NEOLP patients, colored by cell clusters. (**b**) Heatmap of top differentially expressed genes in each cell cluster). (**c**) Heatmap of the ssGSEA enrichment scores for seven senescence-related gene sets. Higher ssGSEA enrichment scores are depicted in red, and genes with lower expression are depicted in blue. (**d**) Violin plot of mean ssGSEA enrichment scores for seven senescence-related gene sets in each cell cluster and disease. UMAP, uniform manifold approximation and projection; EOLP, erosive oral lichen planus; NEOLP, non-erosive oral lichen planus; ssGSEA, single-sample gene set enrichment analysis

**Fig. 3:**
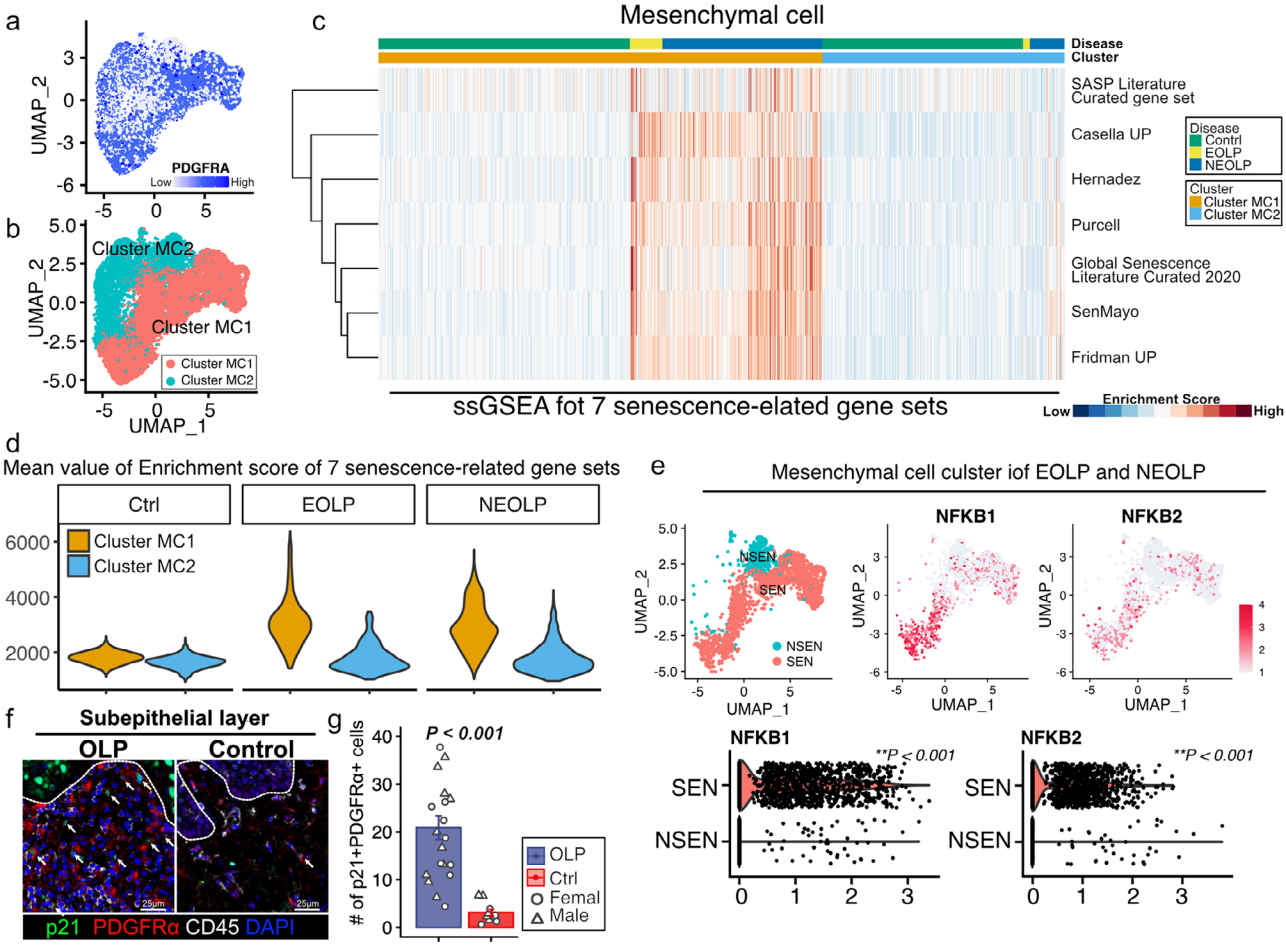
Identification of senescent mesenchymal cells in oral lichen planus (OLP). (**a**) UMAP plot of mesenchymal cells from control, EOLP, and NEOLP patients colored according to PDGFRA expression. (**b**) UMAP plot of mesenchymal cells from control, EOLP, and NEOLP patients., colored by cluster. (**c**) Heatmap of the ssGSEA enrichment scores for seven senescence-related gene sets. Higher ssGSEA enrichment scores are depicted in red, and genes with lower expression are depicted in blue. (**d**) Violin plot of mean ssGSEA enrichment scores for seven senescence-related gene sets in each cell cluster and disease. (**e**) UMAP plot of mesenchymal cells from EOLP and NEOLP patients colored by senescent cluster and feature plot of *NFKB1* and *NFKB2*. Quantification of the gene expression of *NFKB1* and *NFKB2* in each cluster. (**f**) Representative images of immunohistochemical staining for p21, PDGFRα, and CD45 in the subepithelial layer of oral mucosa sections from OLP and control groups. (**g**) Quantification of p21 + PDGFRα + cells in the subepithelial layer. Data are presented as mean and standard error (SE) with dot plots. The circular dot plots represent females, and the triangles represent males. P-values were determined using “FindMarkers” function of Seurat for single-cell RNA-seq data and two-tailed Student’s t-test for immunohistochemical staining UMAP, uniform manifold approximation and projection; EOLP, erosive oral lichen planus; NEOLP, non-erosive oral lichen planus; ssGSEA, single-sample gene set enrichment analysis

We performed scRNA-seq data analysis using the GSE211630 dataset in the NCBI GEO database to identify the cell types that showed senescence. The datasets included buccal mucosal tissue harvested from one healthy control, three patients with non-erosive OLP (NEOLP), and two patients with erosive OLP (EOLP). Ten distinct cell clusters were annotated using uniform manifold approximation and projection (UMAP) (Fig. 2a, Supplementary Fig. 1). Highly expressed genes are shown in the heatmap of each cell cluster (Fig. 2b). Single-sample GSEA (ssGSEA) was performed to identify senescence-related gene expression. The Global Senescence Literature Curated 2020, SASP Literature Curated gene set, SenMayo, Fridman_Up, Purcell, Casella_Up, and Hernadez senescence-related gene sets were used for ssGSEA [26–31]. We found that mesenchymal cell clusters highly expressed senescence-related gene sets in EOLP and NEOLP in the heatmap and violin plot, which showed a mean enrichment score of seven senescence-related gene sets (Fig. 2c, d). To further analyze mesenchymal cell clusters, we performed a sub-cluster analysis. We confirmed that the mesenchymal cell clusters expressed PDGFRA, a marker of mesenchymal cells. UMAP showed eight distinct clusters, and ssGSEA revealed that clusters 0, 1, 2, and 3 in EOLP and NEOLP indicated higher expression of senescence-related gene sets (Supplementary Fig. 2a, b). The cluster with higher expression of senescence-related gene sets was annotated as cluster MC1, and the cluster with lower expression of senescence-related gene sets was annotated as cluster MC2 (Fig. 3b). The heatmap and violin plot of ssGSEA showed that cluster MC1 of EOLP and NEOLP indicated higher expression of senescence-related gene sets, and cluster MC1 of control and cluster MC2 of control, EOLP, and NEOLP indicated lower expression of senescence-related gene sets (Fig. 3c, d). Based on these results, we defined cluster MC1 of EOLP and NEOLP as senescent cell (SEN) clusters and cluster MC1 of control and cluster MC2 of control, EOLP, and NEOLP as non-senescent cell (NSEN) clusters. The SEN cluster also increased the expression of *NFKB1* and *NFKB2*, which are triggers of the SASPs (Fig. 3e) [32]. We also confirmed the presence of senescent mesenchymal cells by IHC analysis, and p21^CIP1/WAF1^ and PDGFRα double-positive cells were increased in the subepithelial layer of patients with OLP (Fig. 3f, g). p16^INK4A^, another senescence marker, was also increased in the OLP subepithelial layer (Supplementary Fig. 3a, b).

### Ligand-receptor analysis of senescent and non-senescent mesenchymal cells in patients with OLP

**Fig. 4:**
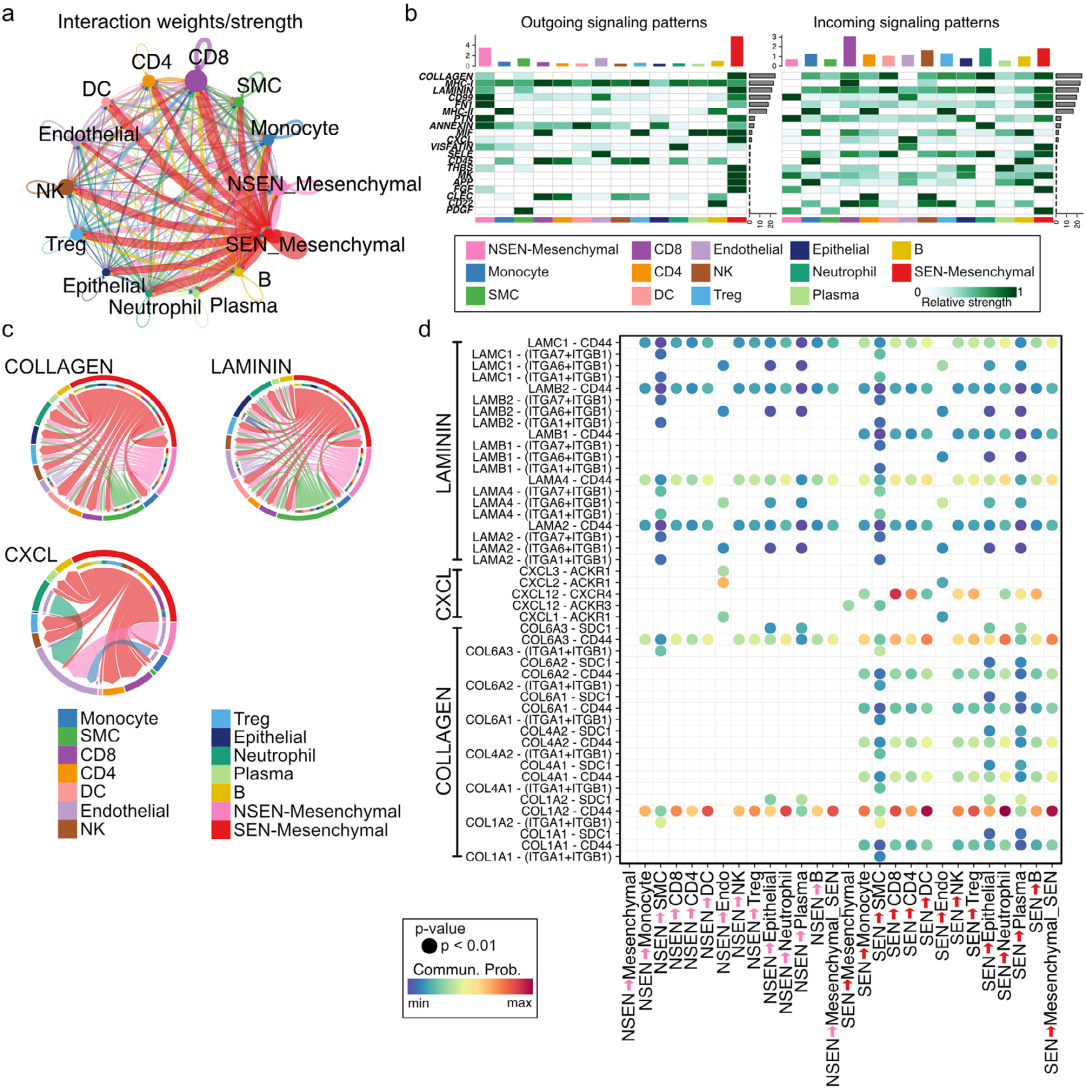
Senescent mesenchymal cells have higher cell − cell communications with immune cells. (**a**) Circles plot significant communication in each cell cluster. The thicker line indicates strong communication. (**b**) Heatmap of the top 20 outgoing/incoming signaling patterns in each cell cluster. (**c**) Chord diagram of the significant network of COLLAGEN, LAMININ, and CXCL signaling pathways in each cell cluster. (**d**) Bubble plot of ligand-receptor pairs in mesenchymal cell clusters. The colors in the bubble plot are proportional to the communication probability, where blue and red correspond to the smallest and largest values, respectively

Using newly defined cell clusters, including SEN_Mesenchymal, NSEN_Mesenchymal, Monocyte, SMC, CD8, CD4, DC, Endothelial, NK, Treg, Epithelial, Neutrophil, Plasma, and B cell clusters, we performed ligand-receptor analysis using CellChat [33]. Ligand-receptor analysis showed that the SEN_Mesenchymal cluster had higher cell-cell communication (Fig. 4a). In-depth analysis showed the top 20 ligand-receptor signaling pathways and the strength of outgoing/incoming signaling in each cell cluster (Fig. 4b). The data showed that the SEN_Mesenchymal cluster was the most expressed outgoing signal, and the CD8 cluster was the most expressed incoming signal (Fig. 4b). NK and Neutrophil clusters also showed high expression of incoming signals (Fig. 4b). We next investigated the dominant signals in the SEN_Mesenchymal cluster instead of the NSEN_Mesenchymal cluster and found that COLLAGEN, LAMININ, and CXCL signals were dominant (Fig. 4b, c). The bubble plot showed significantly increased cell-cell communication in NSEN_Mesenchymal and SEN_Mesenchymal cell clusters, and the data revealed that COL1A1-CD44, COL4A1-CD44, COL4A2-CD44, COL6A1-CD44, COL6A2-CD44, LAMB1-CD44, and CXCL12-CXCR4 were significantly higher in the communication between SEN_Mesenchymal cells, epithelial cells, and immune cells, including the CD4, CD8, NK, Monocyte, Treg, Neutrophil, Plasma, and B cell clusters (Fig. 4d). These results indicate that senescent mesenchymal cells may strongly affect other cell populations.

### Immune cells were increased in the senescent mesenchymal cells accumulated region

**Fig. 5:**
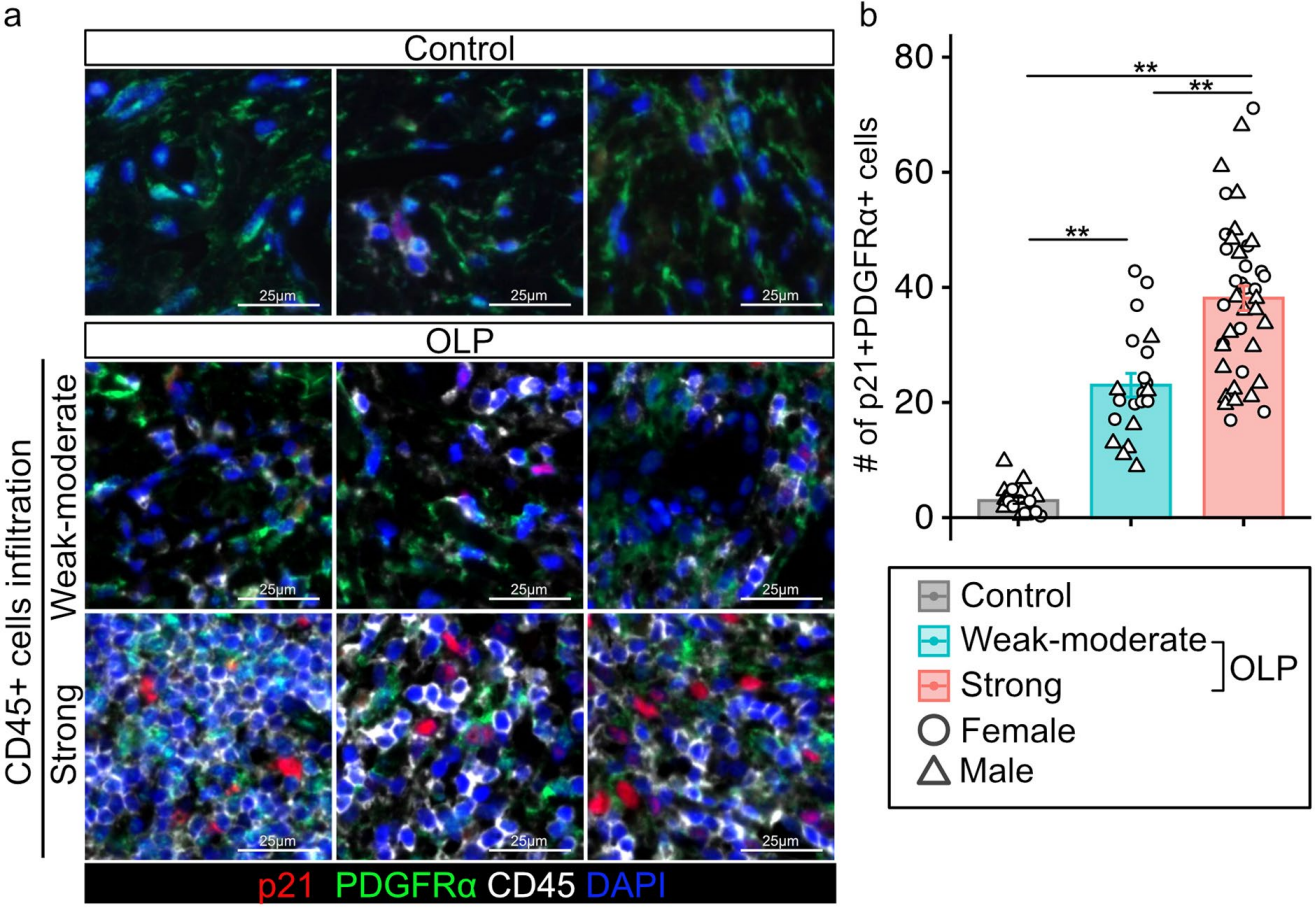
Senescent mesenchymal cell count increased in the strongly immune cell–infiltrated regions. (**a**) Representative images of immunohistochemical staining for p21, PDGFRα, and CD45 in the subepithelial layer of oral mucosa sections from patients with oral lichen planus (OLP) and control groups. CD45-positive immune cell infiltration was graded as weak-moderate (≤ 40% area of CD45-positivity) and strong (> 40% area of CD45-positivity). (**b**) Quantification of p21 positive cells in control and patients with OLP. Data are presented as the mean and standard error (SE) with dot plots. The circular dot plots represent females, and the triangles represent males. *P*-values were determined using Tukey’s method for one-way analysis of variance (**p < 0.001)

Previous studies have shown that accumulated senescent cells contribute to chronic inflammation via excessive immune cell infiltration through SASP expression [34]. Excessive immune cell infiltration into the subepithelial layer is also a histopathological characteristic of OLP [3, 12]. We performed an IHC analysis to investigate whether senescent mesenchymal cells increased in regions with high immune cell infiltration. We found that the extent of highly CD45 + immune cell–infiltrated regions increased in p21-positive mesenchymal cells compared to that in both of Ctrl tissue and weak to moderate immune cell–infiltrated regions (Fig. 5a, b).

### Senescent mesenchymal cell-derived SASP induces epithelial cell senescence and NK and T cell proliferation and activation in vitro

**Fig. 6:**
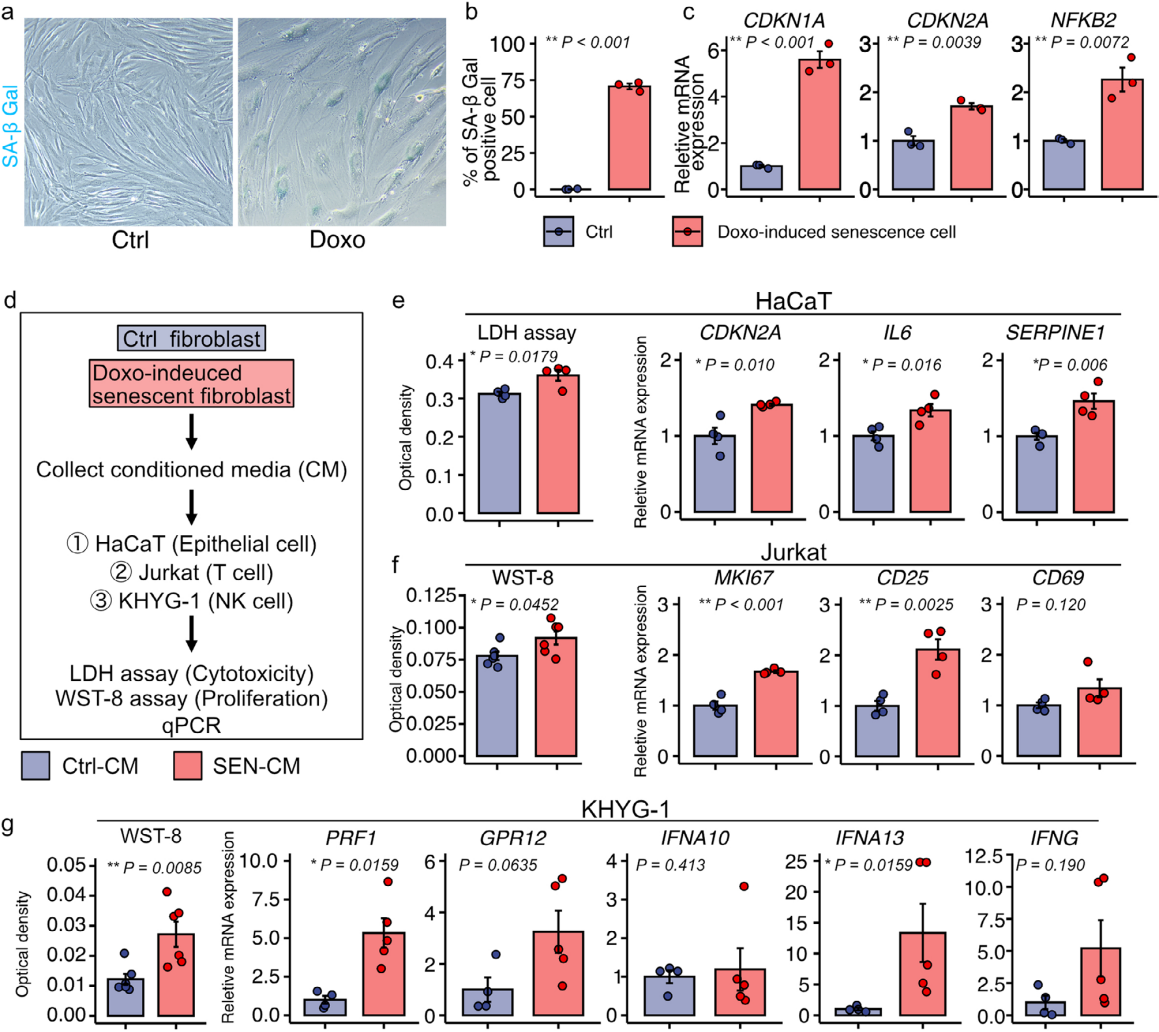
Senescent mesenchymal cell-derived SASP induces epithelial cell senescence and NK and T cell proliferation and activation in vitro. (**a**) Representative images of SA-β-Gal expression in TIG-118 cells with or without doxorubicin (DOXO)-treatment. (**b**) Quantification of SA-β-Gal-positive cells. (**c**) Relative mRNA expression of *CDKN1A*, *CDKN2A*, and *NFKB2* in control and DOXO-induced senescent cells. (**d**) Protocols for in vitro study. (**e**) Quantification of LDH cytotoxicity and relative mRNA expression of CDKN2A, IL6, and SERPINE1 in HaCaT cells treated with control cell-derived conditioned media (Ctrl-CM) and senescent cell-derived conditioned media (SEN-CM). (**f**) Quantification of WST-8 proliferation and relative mRNA expression of MKI67, CD25, and CD69 in Ctrl-CM- and SEN-CM-treated Jurkat cells. (**g**) Quantification of WST-8 proliferation and relative mRNA expression of PRF1, GPR12, IFNA10, IFNA13, and IFNG in Ctrl-CM or SEN-CM-treated KHYG-1 cells. Data are presented as mean and standard error (SE) with dot plots. *P*-values were determined using a two-tailed Student’s t-test. (*P < 0.05 and **P < 0.01) NK, natural killer; SASP, senescence-associated secretory phenotype

To investigate whether senescent mesenchymal cells contribute to OLP pathology, we performed an in vitro assay using senescent mesenchymal-derived SASP-containing conditioned media. First, we confirmed that TIG-118 expressed PDGFRα, a mesenchymal cell marker (Supplementary Fig. 4). Doxorubicin (DOX) was used to induce senescence. The DOXO-treated TIG-118 increased the percentage of senescence-associated beta-galactosidase (SA-β-Gal)-positive cells and the expression levels of *CDKN1A*, *CDKN2A*, and *NFKB2* (Fig. 6a-c), which triggers CXCL12 [35]. Next, to investigate the effect of the SASP on epithelial and immune cells, we collected conditioned media from control TIG-118 and DOX-induced senescent TIG-118 cells. HaCaT, a human skin-derived epithelial cell line; Jurkat, a human T cell line; and KHYG-1, a human NK cell line, were exposed to the conditioned media (Fig. 6d). From scRNA-seq data analysis and GSEA analysis for KEGG NATURAL KILLER CELL-MEDIATED CYTOTOXICITY and GOBP NATURAL KILLER CELL ACTIVATION using GSE38616 and GSE52130 datasets (Supplementary Fig. 5), we focused on T cells and NK cells. Senescent TIG-118-derived conditioned media (SEN-CM)-exposed HaCaT cells showed a significant increase in lactate dehydrogenase (LDH) release and expression of CDKN2A, IL6, and SERPINE1, which are senescence and SASP markers, compared to control TIG-118-derived conditioned media (Ctrl-CM) (Fig. 6e). SEN-CM-exposed Jurkat cells showed increased cell proliferation by assessing WST-8 assay and MKI67 mRNA expression and increased the mRNA expression of CD25, whose expression level was increased in proliferating Jurkat cells (Fig. 6f) [36]. In addition, SEN-CM-exposed KHYG-1 cells also showed increased cell proliferation and mRNA expression of PRF1 and IFNA13, which encode perforin and type I IFN associated with NK cell-mediated cytotoxicity (Fig. 6g). These results suggest that senescent mesenchymal cells have the potential to contribute to OLP pathology by inducing epithelial cell damage and senescence, as well as T cell and NK cell proliferation and activation.

## Discussion

This study revealed that senescent mesenchymal cells are associated with OLP pathology; they activate immune cells, including T cells and NK cells, and induce senescence and cytotoxicity in epithelial cells.

Several studies have reported increased p21^CIP1/WAF1^, p16^INK4A^, and p53-positive cells in patients with OLP by IHC analysis [23–25]. Other studies showed that SMP-30, a marker of senescence and anti-apoptosis, was upregulated in OLP [37, 38]. Although these findings indicate a relationship between senescence and OLP pathogenesis, using a single senescence marker is insufficient to detect senescent cells, and applying a validated senescence gene set is considered a viable method for detecting senescence in vivo [27]. Our study analyzed seven validated senescence gene sets and two transcriptome data cohorts of patients with OLP from GEO to explore the association between senescence and OLP. The results of GSEA indicated significant enrichment of senescence gene sets in patients with OLP, confirming the possibility of a connection between senescence and OLP.

scRNA-seq and IHC analyses of patients with OLP revealed that senescence is exhibited by mesenchymal cells located in the subepithelial layer^36^. We utilized PDGFRα as a marker for mesenchymal cells or fibroblasts. There is significant documentation of the features of senescent PDGFRα-positive cells in various organs, such as the skin, skeletal muscle, lung, and adipose tissue [39–43]. These studies reported that senescent PDGFRα-positive cells exhibited increased SASP expression, which can promote the recruitment of immune cells. In the scRNA-seq analysis, we found that the mesenchymal cell cluster, which expressed *PDGFRA*, increased senescence-related gene expression in *EOLP* and *NEOLP* but not in the control tissue. Using CellChat, a ligand-receptor analysis tool, we found that senescent mesenchymal cell clusters strongly interact with immune cells, especially CD8 + T cells and NK cells. CellChat analysis also showed that senescent mesenchymal cell clusters have significant connections with immune cells via CXCL12-CXCR4. CXCL12 serves as ligands for CXCR4, a receptor that plays an important role in activating and homing CD8 + T cells and NK cells towards CXCL12-expressing cells [44–46]. Our findings suggest that augmented CXCL12-CXCR4 signaling in senescent mesenchymal cells may cause the accumulation of CD8 + T cells and NK cells in the vicinity of senescent mesenchymal cells in patients with OLP.

In the present study, the scRNA-seq analysis revealed that senescent mesenchymal cells are connected to immune cells via collagen IV, VI-CD44, and laminin-CD44. CD44 is a cellular adhesion molecule expressed in many different cell types and a receptor for hyaluronic acid, laminin, collagen, or fibronectin [47, 48]. CD44 promotes the migration of many cell types, including T cells and NK cells, by regulating their adhesion and extravasation [49]. CD44-positive cell-matrix interactions promote cell proliferation, adhesion, migration, and lymphocyte activation [50]. Although the functional relationship between senescent mesenchymal cells generating collagen, laminin, and CD44 remains unclear, the cell-matrix interaction with CD44 may be related to the OLP pathomechanism.

In our in vitro experiments, we explored the effects of senescent cells on immune and epithelial cells using a SASP-containing conditioned media (SEN-CM) isolated from senescent fibroblasts. Our findings indicate that SEN-CM stimulates the proliferation of Jurkat cells and augments the expression of the cellular proliferation markers MKI67 and CD25 [36]. SEN-CM also activates KHYG-1 cells, which are cytotoxic to NK cells [51]. SEN-CM treatment increased the expression of PRF1, which encodes perforin, and GPR12, which regulates the cytolytic activity of NK cells [52]. Moreover, SEN-CM increased the cytotoxicity of HaCaT cells, an epithelial cell line, and upregulated the expression of CDKN2A, IL6, and SERPINE1, which are typical markers of senescence. These findings suggest that senescent mesenchymal cell-derived SASP may contribute to OLP pathology by activating T cells and NK cells and inducing epithelial damage and senescence.

Although OLP is a T cell-mediated inflammatory disease of the oral mucosa, this study identified an NK cell population that may be associated with OLP pathology based on scRNA-seq analysis and GSEA. GSEA revealed significant enrichment of KEGG NATURAL KILLER CELL-MEDIATED CYTOTOXICITY and GOBP NATURAL KILLER CELL ACTIVATION in patients with OLP. The role of NK cells in OLP pathogenesis remains unclear; however, previous studies have reported that NK cells constituted 2.3–15% of cellular infiltrates in cutaneous lichen planus [14, 53]. Therefore, NK cells may provide an early stimulatory signal for the mobilization of CD4 + and CD8 + T cells upon their arrival in lichen planus-inflamed areas [54]. Our data from the bioinformatics analysis suggest the possibility of NK cell involvement in OLP pathogenesis, which may support the hypothesis mentioned above.

Senescent cells are implicated in the pathogenesis of periodontal diseases and oral submucous fibrosis [55, 56]. In periodontal disease, persistent infection with Gram-negative bacteria leads to DNA damage and subsequent senescence of host cells, resulting in chronic inflammation [55]. In oral submucous fibrosis, senescence occurs in keratinocytes, fibroblasts, and endothelial cells induced by exposure to areca nut alkaloids, causing DNA damage and increased ROS levels [56, 57]. While senescence is a known suppressor of oral squamous cell carcinoma (OSCC) development owing to permanent cell cycle arrest, senescence of the non-epithelial components, especially cancer-associated fibroblasts (CAFs), contributes to OSCC development [58]. CAFs are critical components of the tumor microenvironment with diverse functions, including matrix deposition and remodeling, and extensive reciprocal signaling between cancer cells to promote cancer progression [59]. CAFs originate from normal fibroblasts, pericytes, smooth muscle cells, or mesenchymal cells [60]. Further investigation is necessary to determine whether senescent mesenchymal cells in OLP contribute to tumor suppression or development, which may help in the development of senescent cell-targeting therapies for OLP.

There are several limitations in this study. First, human skin fibroblast (TIG-118) was used in our in vitro experiments. Fibroblasts are thought to play an important role in wound healing and scar formation and oral mucosal fibroblasts proliferate slightly more than skin fibroblasts [61]. We also used HaCaT cells which are a spontaneously immortalized keratinocyte cell line derived from adult human skin. HaCaT cells were previously used in oral cell biology as an oral keratinocyte model [62]. However, keratinocytes from the skin and oral mucosa have differences in morphology, gene expression, and proliferative capacity [63]. These differences may have affected the results of our in vitro experiments. Second, this study showed more CD45 + cells with senescent mesenchymal cells and CD8+/NK proliferation and secretory function. Although cell–cell communication analysis using scRNA-Seq revealed that senescent mesenchymal cells significantly influenced CD8 + T and NK cells, further experiments are needed to elucidate the details of the induction and migration of CD8 + T and NK cells by senescent mesenchymal cells in OLP pathology. Third, we used healthy regions from patients with leukoplakia as controls, acquired as part of the usual diagnostic procedure. Previous research used healthy tissue and healthy regions from patients with leukoplakia as controls [64]. The study showed that healthy tissue and healthy regions from patients with leukoplakia have significant differences from leukoplakia and OSCC tissues, as determined using histological and mechanical evaluations by measuring tissue stiffness and the expression levels of p16 and BCL-2, which showed that healthy regions from patients with leukoplakia could be used as a control [64]. Although healthy regions from patients with leukoplakia can be used as a control, healthy tissue could also be analyzed as a control.

**Fig. 7:**
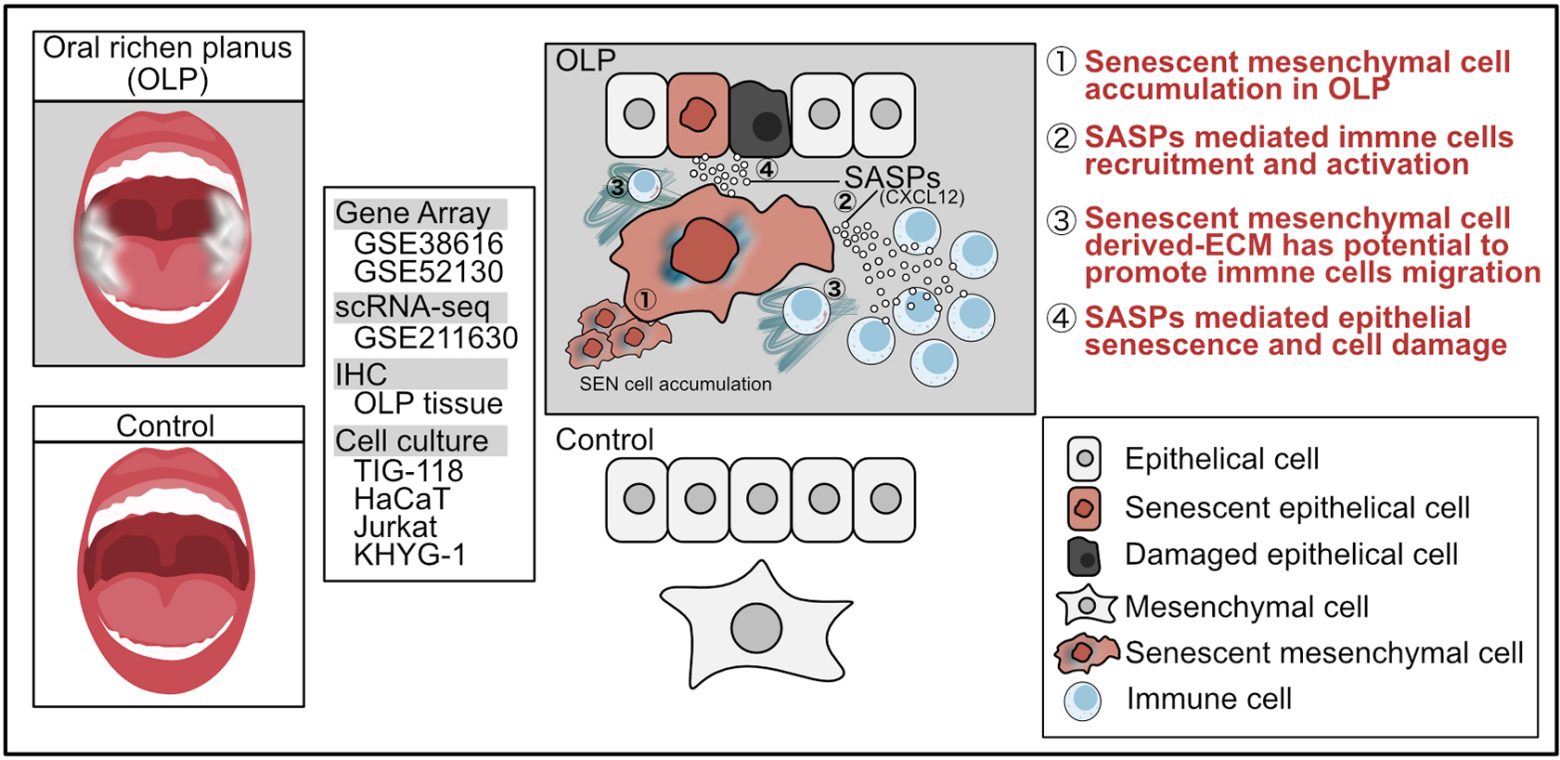
Overview of key findings

In conclusion, this study indicates that senescent mesenchymal cells are associated with OLP pathology by activating immune cells, including T cells and NK cells, and inducing senescence and cytotoxicity in epithelial cells (Fig. 7). These findings reveal a new pathological mechanism of OLP, which is mediated by senescence, and provide potential therapies targeting senescent cells.

## Materials and methods

### GEO data

Gene expression data for the OLP and control oral tissue samples were obtained from the NCBI GEO using the GEOquery R package [65]. The accession numbers of the datasets are GSE38616 and GSE52130. Both datasets included seven OLP and seven control samples. scRNA-seq dataset GSE211630 was obtained from the NCBI GEO. The dataset included the buccal mucosa from two EOLP patients, three NEOLP patients, and one healthy control tissue [15].

### GSEA

GSEA was performed using the GSEA software (https://www.gsea-msigdb.org/gsea/index.jsp), a joint project of the UC San Diego and Broad Institute [66, 67]. GSEA analyses were performed using seven gene sets related to senescence and SASP, including the Global Senescence Literature Curated 2020, SASP Literature Curated gene set, SenMayo, Fridman Senescence Up, Purcell, Casella Up, and Hernandez gene sets [26–31], and two gene sets related to NK cells, including KEGG NATURAL KILLER CELL-MEDIATED CYTOTOXICITY and GOBP NATURAL KILLER CELL ACTIVATION.

### ScRNA-seq analysis

The scRNA-seq dataset GSE211630 was imported into R (version 4.2.2), and Seurat (version 4.3.0) was used [68, 69]. The data were filtered to include genes detected in > 3 cells, cells with 100–5,000 detected genes, and a mitochondrial gene expression ratio of < 5%. The scRNA-seq datasets were normalized using the “NormalizeData” function, and 2000 highly variable genes were identified using the “FindVariableFeatures” function. Then, the canonical correction analysis method with the “FindIntegrationAnchors” and “IntegrateData” functions was used to remove batch effects. Next, we performed data scaling, used the PCA method to reduce the dimensions, and used the UMAP to visualize the data. Cell clusters were identified using the “FindNeighbors” function and the “FindClusters” function with a resolution of 0.5. We utilized already known marker genes to annotate cell clusters: mesenchymal cell (PDGFRA), smooth muscle cell (SMC) (ACTA2), endothelial cell (VWF), epithelial cell (KRT5), neuron (NRXN1), skeletal muscle (DES), immune cells (PTPRC), T cell (CD3E), CD4 + T cell (CD4), regulatory T cell (Treg) (FOXP3), CD8 + T cell (CD8A), NK cell (GNLY), monocyte (AIF1), Dendritic cell (DC) (FCER1A), plasma cell (MZB1), neutrophil (S100A8), and B cell (MSA4A1).

### ssGSEA

ssGSEA was performed using the Easy single-cell analysis platform for enrichment (version 1.8.0) [70]. Enrichment scores were calculated for each of the seven senescence-related gene sets, including the Global Senescence Literature Curated 2020, SASP Literature Curated gene set, SenMayo, Fridman_Up, Purcell, Casella_Up, and Hernadez senescence-related gene sets [26–31], and visualized in a heatmap and violin plot using dittoSeq (version 1.10.0) [71].

### Cell-cell communication analysis based on ligand-receptor interaction

CellChat, which quantitatively infers and analyzes intercellular communication networks from scRNA-seq data, was used for cell-cell communication analysis based on ligand-receptor interaction (version 1.6.1) [33]. CellChat assigns a communication probability value based on the law of mass action and the average expression values of a ligand by one cell cluster and that of a receptor by another cell cluster. The statistical significance of communication probability values was assessed using a permutation test. *P* < 0.05 was considered significant.

### Patients and tissue samples

The study protocol was approved by the Institutional Review Board of Sapporo Medical University (approval number: 332 − 153).

Twenty patients diagnosed with OLP at the Department of Oral Surgery, Sapporo Medical University, were enrolled in this study, including 10 males and 10 females aged 41–89. Healthy regions from patients with leukoplakia, including five males and five females aged 44–71 years, were used as controls. Hematoxylin and eosin staining images are shown in Supplementary Fig. 6.

### Immunohistochemistry

Paraffin-embedded tissues were sectioned (3-µm thickness). Sections were deparaffinized and rehydrated for immunostaining. Antigen retrieval was performed in a microwave oven (95–98°C for 10 min) using a citrate buffer (10 mM sodium citrate, pH 6.0). After cooling, the slides were washed twice with deionized water and quenched with 3% hydrogen peroxide for 10 min. Then sections were blocked with 1% bovine serum albumin in Tris-buffered saline containing Tween-20 (TBST) for 15 min at room temperature (RT: 20–25°C) and then incubated with primary antibodies overnight at 4°C or for 1 h at RT. After washing three times with TBST for 5 min each, the sections were incubated with SignalStain Boost IHC Detection Reagent (Cell Signaling Technology, MA, USA) for 30 min at RT in the dark. The sections were then washed in TBST three times for 5 min each and treated with TSA Plus Working Solution (Fluorescein, Cyanine 3, and Cyanine 5; Akoya Biosciences, MA, USA) for 10 min at RT in the dark. For multiplex staining, stripping was performed in a microwave oven (95–98°C for 10 min) using citrate buffer. After cooling, the cells were stained with different tyramide fluorescent labels, as described above. The following antibodies were used: anti-CD45 (ab8216, mouse monoclonal, 1:1,000; Abcam, Cambridge, UK), anti-p21 (ZBR1141, rabbit monoclonal, 1:1,000; Sigma-Aldrich, MO, USA), anti–PDGFRα (#3174, 1:1,000; Cell Signaling Technology), and anti–p16INK4A (ZBR1437, rabbit monoclonal, 1:200; Sigma-Aldrich). Nuclei were stained with 4’,6-diamidino-2-phenylindole (Dojindo Laboratories, Kumamoto, Japan). The sections were observed under a fluorescence microscope (Axio Observer 7; ZEISS, Baden-Wurttemberg, Germany). Images of randomly selected epithelial and subepithelial regions were acquired, and the average value of three fields per patient was used for statistical analysis. The fluorescence intensities of p21 and p16INK4A were analyzed using the image analysis function of ZEN (ZEISS). CD45-positive immune cell infiltration was graded as weak-moderate (area of CD45-positivity is 40% or less) and strong (area of CD45-positivity is more than 40%) [34].

### Cell culture

TIG-118, a human skin fibroblast (JCRB cell bank: JCRB0535); HaCaT, a human skin epithelial cell line (Sapporo Medical University Cell Bank); Jurkat, a human T cell line (RIKEN cell bank: RCB3052); and KHYG-1, a human NK cell line (JCRB cell bank: JCRB0156) were used for the in vitro study. TIG-118 and HaCaT cells were cultured in Dulbecco’s modified Eagle’s medium (DMEM) containing 10% fetal bovine serum (FBS). Jurkat and KHYG-1 cells were cultured in RPMI1640 medium containing 10% FBS, and IL-2 was added to the KHYG-1 cells to promote cell proliferation.

### Western blotting

Cell pellets were lysed in a cell lysis buffer (Nacalai Tesque Inc., Kyoto, Japan) with a protease and phosphatase inhibitor cocktail (Nacalai). All lysates were centrifuged, and proteins were quantified using the BCA protein assay (Thermo Fisher Scientific, MA, USA). Then, 8 µg of protein from each sample was electrophoresed on 4–12% Bris-Tris protein gels and transferred to low-fluorescence PVDF membranes (Bio-Rad Laboratories, CA, USA). Blots were probed with the following primary antibodies: anti-PDGFRα (1:1000, Cell Signaling Technology), anti-β actin (1:1000, MEDICAL&BIOLOGICAL LABORATORIES (MBL), Tokyo, Japan), and anti-α tubulin (1:1000, MBL). The blots were probed with anti-rabbit IgG (IRDye® 800CW, 1:20000, LI-COR Biosciences, NE, USA) and anti-mouse IgG (IRDye® 680RD, 1:20000, LI-COR) secondary antibodies. Fluorescence-based detection was performed using LI-COR Odyssey XF (LI-COR).

### SA-β-Gal staining

To detect cellular senescence, we performed SA-β-Gal staining using the Senescence b-Galactosidase Staining Kit (Cell Signaling Technology). Cells were observed using an inverted microscope (Primovert, ZEISS), and the percentage of SA-β-Gal-positive cells was calculated by dividing the number of SA-β-Gal-positive cells by the total number of cells observed.

### Senescence induction and collection of conditioned media

TIG-118 cells were seeded in culture flasks and cultured overnight in DMEM supplemented with 10% FBS. The next day, the complete medium was replaced with 250 nM DOX-containing complete medium and incubated for 24 h to induce senescence. After DOX treatment, TIG-118 cells were washed with phosphate-buffered saline and cultured in a fresh complete medium. Eight days after DOX treatment, the complete medium was replaced with a low-serum medium containing 0.2% FBS and incubated for 48 h. After 48 h, the medium was replaced with a serum-free and phenol-red-free medium, and the cells were incubated for 24 h. After 24 h, the SASP-containing conditioned media was collected. The collected conditioned media was centrifuged, and the supernatants were stored in a -80 °C refrigerator for subsequent experiments.

### Cell proliferation and cytotoxicity lactate dehydrogenase assay

HaCaT, Jurkat, and KHYG-1 cells were cultured in conditioned media supplemented with 2% FBS for 48 h. After 48 h, cell proliferation or cytotoxicity lactate dehydrogenase assay was performed using the Cell Counting Kit-8 (Dojindo) or Cytotoxicity LDH Assay kit (Dojindo).

### RNA extraction and quantitative real-time PCR

Total RNA was isolated from HaCaT, Jurkat, and KHYG-1 cells cultured in a conditioned media containing 2% FBS for 48 h using TRI Reagent (Molecular Research Center Inc., OH, USA). The isolated RNA was reverse-transcribed into cDNA using the iScript Advanced cDNA Synthesis Kit (Bio-Rad). Quantitative PCR was performed using the SsoAdvanced Universal SYBR Green Supermix (Bio-Rad) in a CFX Connect Real-Time PCR Detection System (Bio-Rad) under the following cycling conditions: 95 °C for 30 s, followed by 40 cycles of amplification (95 °C for 10 s and 60 °C for 30 s). The primer sequences used for the PCR are listed in Supplementary Table 1. The samples were compared using the ΔΔCt method.

### Statistical analysis

Quantitative data are reported as means and standard errors (SE). Each data point was plotted using dot and violin plots with ggplot2, a plotting system for R based on the Grammar of Graphics. Normality was assessed using the Shapiro–Wilk test. Pairwise t-tests were used to compare two groups. One-way analysis of variance was conducted to assess differences between three or more groups. *P*-values for multiple comparisons were adjusted using Tukey’s method. Statistical analyses were performed using EZR, a graphical user interface for R [72]. Two-sided *P*-values less than 0.05 were considered statistically significant. For the statistical analysis of GSEA, the Singal2Noise method, a ranking method based on t-statistics, was used to calculate the *P*-value, and the false discovery rate method was used to calculate the q-value.

### Electronic supplementary material

Below is the link to the electronic supplementary material.

**Supplementary Material 1**: **Supplementary Figure 1. **UMAP plot of each cell marker gene. **Supplementary Figure 2.** UMAP plot of mesenchymal cell clusters and ssGSEA. **Supplementary Figure 3. **P16INK4A expression in the subepithelial layer of oral mucosa sections from patients with OLP and controls. **Supplementary Figure 4. **Detection of PDGFRα in TIG-118 cells by western blotting. **Supplementary Figure 5. **NK cells and T cells were activated in OLP. **Supplementary Figure 6. **Hematoxylin and eosin–stained images of samples from patients with oral lichen planus (OLP) and healthy regions from patients with leukoplakia as controls. **Supplementary Table 1. **The primer sequences used for the q-PCR

## Data Availability

Data and materials not indicated in this manuscript are available from the corresponding author.
