# Supplementary material for: Senescence-associated secretory phenotypes in mesenchymal cells contribute to cytotoxic immune response in oral lichen planus
Source: Immun Ageing. 2023 Dec 5;20:72. doi: 10.1186/s12979-023-00400-5 (PMC10696703; doi:10.1186/s12979-023-00400-5)
Supplement: Supplementary file 1 — Supplementary Material 1: Supplementary Figure 1. UMAP plot of each cell marker gene. Supplementary Figure 2. UMAP plot of mesenchymal cell clusters and ssGSEA. Supplementary Figure 3. P16INK4A expression in the subepithelial layer of oral mucosa sections from patients with OLP and controls. Supplementary Figure 4. Detection of PDGFRα in TIG-118 cells by western blotting. Supplementary Figure 5. NK cells and T cells were activated in OLP. Supplementary Figure 6. Hematoxylin and eosin–stained images of samples from patients with oral lichen planus (OLP) and healthy regions from patients with leukoplakia as controls. Supplementary Table 1. The primer sequences used for the q-PCR [file 12979_2023_400_MOESM1_ESM.pdf]

Supplementary File for

Senescence-associated secretory phenotypes in mesenchymal cells contribute to cytotoxic immune response in oral lichen planus

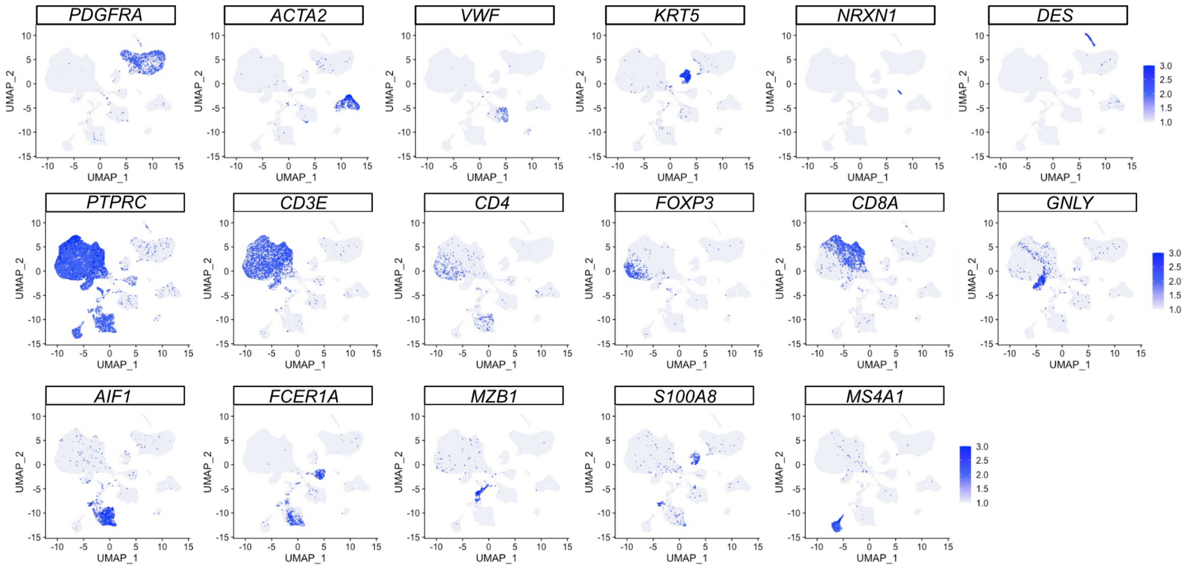

Supplementary Figure 1. UMAP plot of each cell marker gene

UMAP plot of each cell marker gene in buccal mucosa cells from control, EOLP, and NEOLP patients, colored according to the expression level of each gene.

UMAP, uniform manifold approximation and projection; EOLP, erosive oral lichen planus; NEOLP, non-erosive oral lichen planus

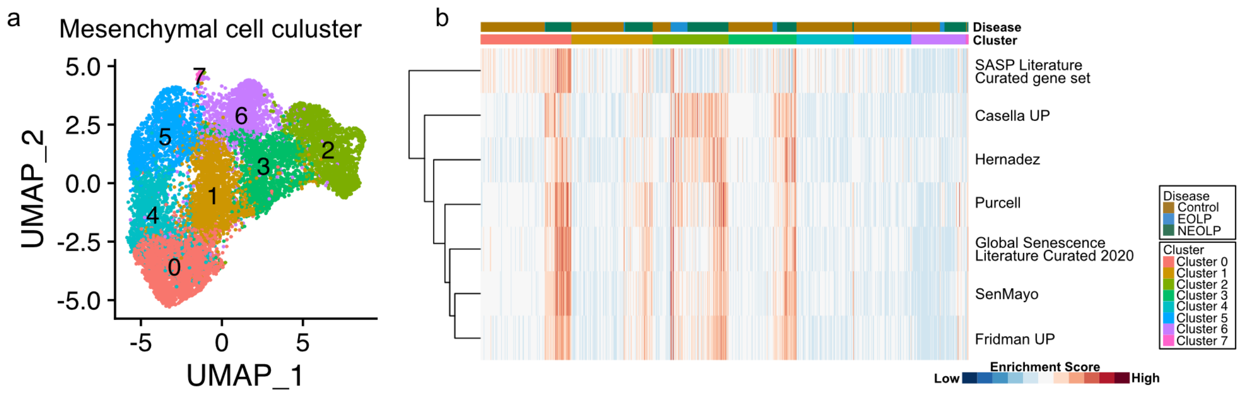

Supplementary Figure 2. UMAP plot of mesenchymal cell clusters and ssGSEA.

(**a**) UMAP plot of mesenchymal cells from control, EOLP, and NEOLP patients, colored by sub-clusters. (**b**) Heatmap of the ssGSEA enrichment scores for seven senescence-related gene sets. Higher ssGSEA enrichment scores are shown in red, and genes with lower expression are shown in blue.

UMAP, uniform manifold approximation and projection; EOLP, erosive oral lichen planus; NEOLP, non-erosive oral lichen planus; ssGSEA, single-sample gene set enrichment analysis

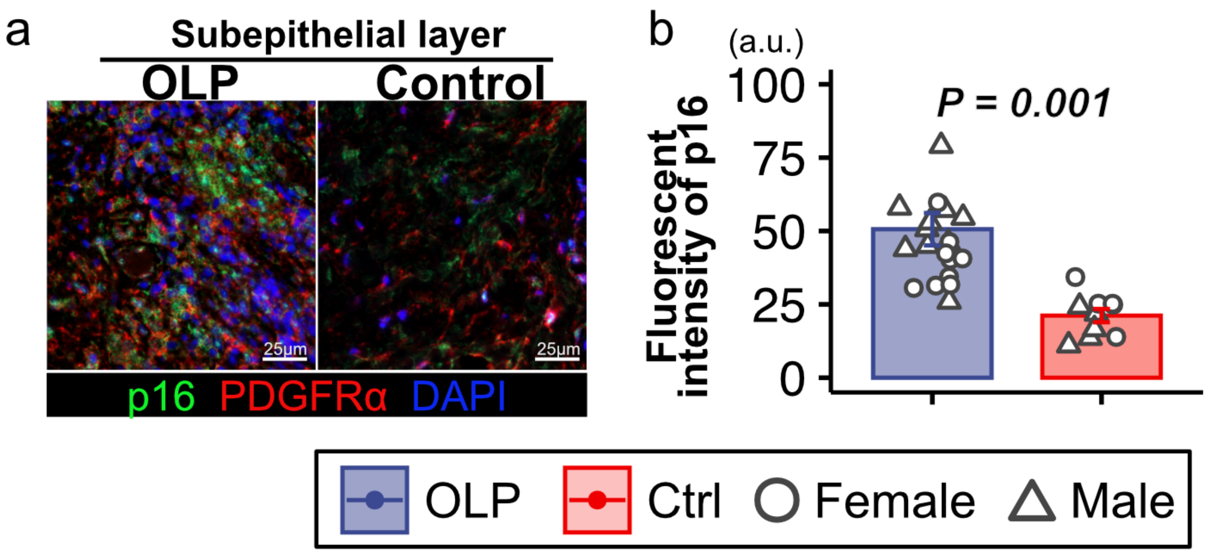

Supplementary Figure 3. P16^{INK4A} expression in the subepithelial layer of oral mucosa sections from patients with OLP and controls

(**a**) Representative images of immunohistochemical staining for p16^{INK4A} and PDGFRα in the subepithelial layer of oral mucosa sections from OLP and control. (**b**) Quantification of p16^{INK4A} expression in the epithelial layer was performed using fluorescence intensity (arbitrary units, a.u.). Data are presented as mean and standard error (SE) with dot plots. The circular dot plots represent females, and the triangles represent males. The P-values were determined using a two-tailed Student's t-test.

OLP, oral lichen planus

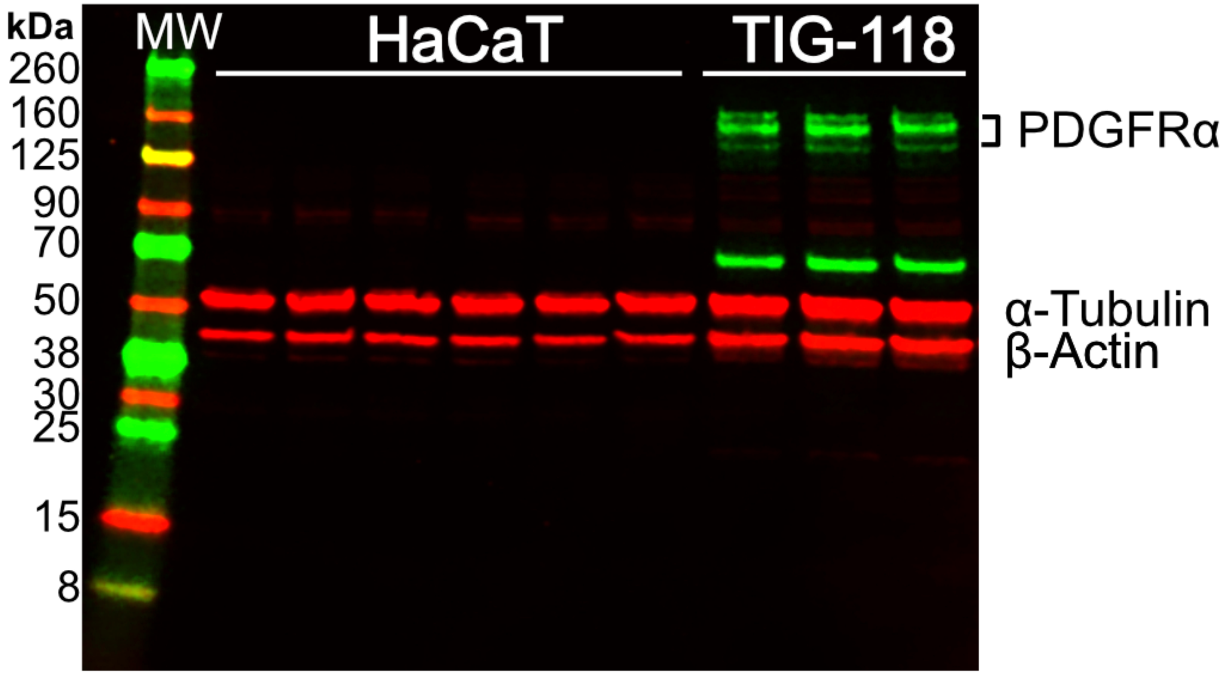

Supplementary Figure 4. Detection of PDGFRα in TIG-118 cells by western blotting.

Representative images of western blotting of HaCaT and TIG-118 cells stained with PDGFRα, α-tubulin, and β-actin.

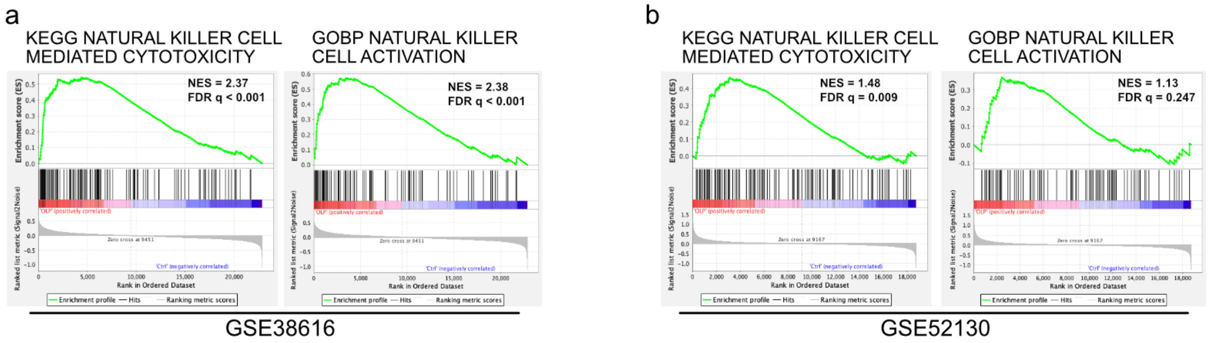

Supplementary Figure 5. NK cells and T cells were activated in OLP.

GSEA for KEGG NATURAL KILLER CELL-MEDIATED CYTOTOXICITY and GOBP NATURAL KILLER CELL ACTIVATION (**c, d**). The normalized enrichment scores and FDR q values are listed in each GSEA plot.

GSEA, gene set enrichment analysis; FDR, false discovery rate; OLP, oral lichen planus

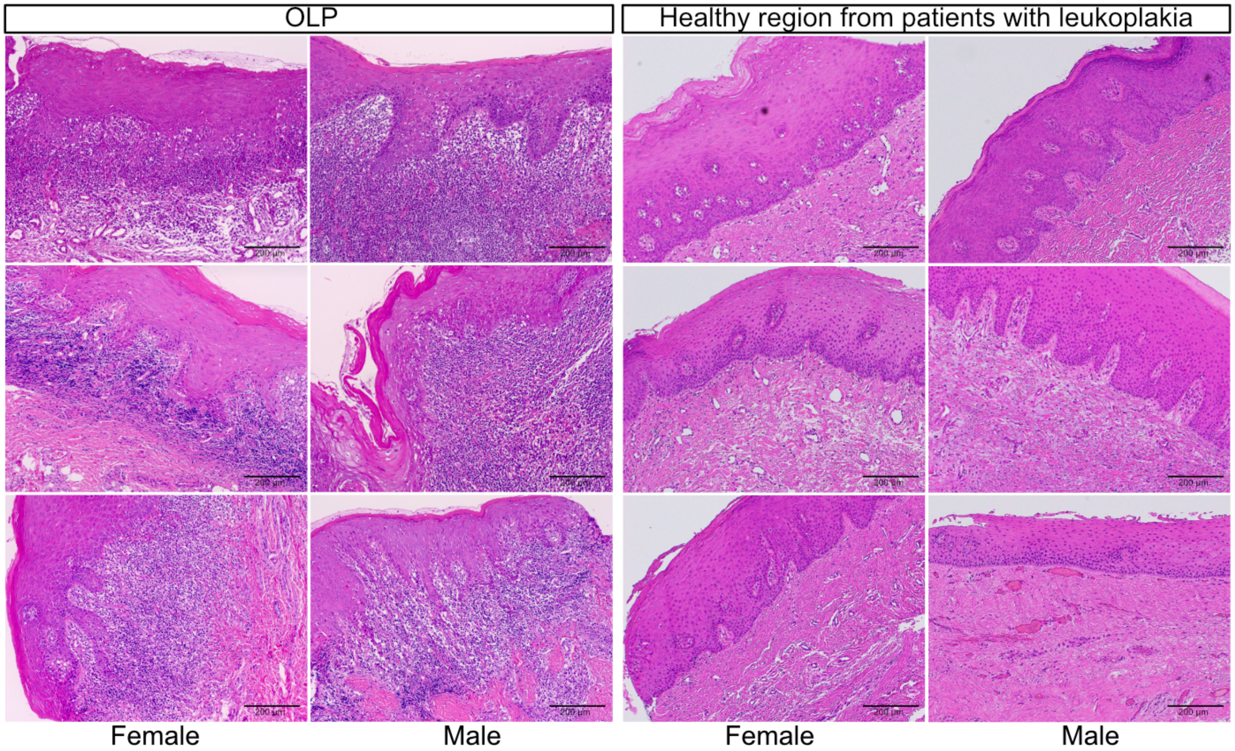

Supplementary Figure 6. Hematoxylin and eosin–stained images of samples from patients with oral lichen planus (OLP) and healthy regions from patients with leukoplakia as controls. Scale bars = 200 µm.

Supplementary Table 1. The primer sequences used for the q-PCR

Gene	Forward	Reverse
CDKN1A	AGGTGGACCTGGAGACTCTCAG	TCCTCTTGGAGAAGATCAGCCG
CDKN2A	ATCATCAGTCACCGAAGGTC	CTCAAGAGAAGCCAGTAACC
NFKB2	GGCAGACCAGTGTCATTGAGCA	CGACCCCTGGAATGTCACAC
TP53	GGACAGCCACGTCTGTGACTTG	CAGCAGAAAGCTCACCACACTC
IL6	GCAGAAAAGGCAAAGAATC	CTACATTTGCCGAAGAGC
SERPINE1	ATCCACAGCTGTCATAGTC	CACTTGGCCCATGAAAAG
MKI67	GAAAGAGTGGCAACCTGCCTTC	GCACCAAGTTTTACTACATCTGCC
CD25	GAGACTTCCTGCCTCGTCACAA	GATCAGCAGGAAAACACAGCCG
CD69	GCTGGACTTCAGCCCAAAATGC	AGTCCAACCCAGTGTTCCTCTC
PRF1	ACTCACAGGCAGCCAACTTTGC	CTCTTGAAGTCAGGGTGCAGCG
GPR12	GCTTCAGTCAGAAGCCACCAAG	TACAGTGAGAGGTAGCGGTCAAC
IFNA10	GTTCCAGAAGGCTCAAGCCATC	TAGGAGGCTCTGTTCCCAAGCA
IFNA13	AGAAGGCTCCAGCCATCTCTGT	TGCTGGTAGAGTTCGGTGCAGA
IFNG	GAGTGTGGAGACCATCAAGGAAG	TGCTTTGCGTTGGACATTCAAGTC
ACTB	AGAGCTACGAGCTGCCTGAC	AGCACTGTGTTGGCGTACAG
Hprt1	CATTATGCTGAGGATTTGGAAAGG	CTTGAGCACACAGAGGGCTACA
TBP	TGTATCCACAGTGAATCTTGGTTG	GGTTCGTGGCTCTCTTATCCTC
GAPDH	ATTGCCCTCAACGACCACTT	TGCTGTAGCCAAATTCGTTGTC
